# Making a living off the rainbow’s edge: How phycobilisomes adapt structurally to absorb far-red light

**DOI:** 10.1016/j.jbc.2024.107262

**Published:** 2024-04-03

**Authors:** Matthew S. Kimber

**Affiliations:** Department of Molecular and Cellular Biology, University of Guelph, Guelph, Ontario, Canada

**Keywords:** allophycocyanin, cryo-electron microscopy, cyanobacteria, far-red light photoacclimation, phycobilisome

## Abstract

Cyanobacteria harvest light by using architecturally complex, soluble, light-harvesting complexes known as phycobilisomes (PBSs). PBS diversity includes specialized subunit paralogs that are tuned to specific regions of the light spectrum; some cyanobacterial lineages can even absorb far-red light. In a recent issue of the *Journal of Biological Chemistry*, Gisriel *et al.* reported the cryo-electron microscopic structure of a far-red PBS core, showing how bilin binding in the α-subunits of allophycocyanin paralogs can modify the bilin-binding site to red shift the absorbance spectrum. This work helps explain how cyanobacteria can grow in environments where most of the visible light has been filtered out.

Cyanobacteria are photosynthetic bacteria that serve as key primary producers in environments as diverse as the open ocean, hot spring microbial mats, and desert crusts. Most cyanobacteria are obligate photoautotrophs and, like higher plants, contain both photosystems I and II (PSI and PSII). However, unlike plants, cyanobacteria (and red algae) use soluble light-harvesting antennae called phycobilisomes (PBSs) (1) to harvest light. PBSs are predominantly comprised of phycobiliproteins (PBPs), of which there are several distinct families, each of which is tuned to absorb a different region of the spectrum. The fundamental building block of PBPs is an αβ heterodimer, with both (distantly related) chains adopting globin folds and interacting through an N-terminal α-helical hairpin motif. These αβ heterodimers assemble into trimeric rings, pairs of which interact to form face-to-face (αβ)_6_ hexamers; higher order assemblies are built by linking two or more such hexamers back to back using linking proteins. In the cell, 2 to 5 bihexameric rods pair lengthwise to form the PBS core, onto which additional peripheral rods assemble endwise (2, 3, 4). This PBS complex sits on the thylakoid membrane and interacts with PSII (more rarely PSI), channeling light energy inward from the PBS periphery to the core, and then to PSII where it ultimately drives the light-mediated reactions (5). The overall molecular mass of a PBS can exceed 7 MDa, and they can collectively comprise up to 50% of the soluble protein mass of a cyanobacterial cell (5, 6).

Cyanobacteria can adapt to the changing spectral qualities of available light by altering the protein composition of their PBSs. An extreme example of this occurs in conditions such as those found in the soil or deep within a microbial mat, where most shorter-wavelength light has been absorbed or scattered. Under these conditions, many cyanobacteria can undergo far-red light photoacclimation (FaRLiP), replacing multiple proteins in PSI, PSII, and PBS with specialized far-red light–adapted paralogs (1, 7); this then allows the cells to grow even on near-infrared light (700–750 nm). In a recent issue of the *Journal of Biological Chemistry*, Gisriel *et al.* (8) reported the single-particle cryo-electron microscopy structure of a PBS from *Synechococcus* sp. PCC 7335 cells grown under far-red light conditions, at 2.78 Å resolution. While not the focus of their study, they also report a 2.35 Å structure for Rubisco, an abundant, well-studied Calvin cycle enzyme that fixes CO_2_ during photosynthesis, which copurified with their particles.

PBPs are a diverse group of proteins grouped into several major families with differing spectral properties. The FaRLiP-adapted PBSs reported by Gisriel *et al.* (5) contain only allophycocyanins (Apcs), which specifically absorb longer wavelength light and appear aqua colored in solution. The cryo-electron microscopy maps allowed the authors to model three complete αβ trimeric rings of Apc subunits; the authors also inferred that a fourth ring, with composition ApcB2_3_ ApcD5_3_ ApcC, occupies the distal end of the complex *in vivo* but is lost during later purification steps. Eight of the visualized nine β-type subunits are copies of ApcB2, with the remaining β-subunit being ApcF. The α-type subunits are more diverse, with six copies of ApcD5 plus one copy each of ApcD2, ApcD3, and ApcE2. In addition to its Apc domain, ApcE2 also contains two repeat domains that localize to the center of the trimeric rings, linking two adjacent rings; the third ring is occupied by ApcC, a stand-alone linker protein. Note that each subunit occupies specific position(s) in the complex; details can be seen in Figure 1, *A*–*D*. Of note, only structures of ApcF and ApcC have been previously reported in context of other PBS structures; the other five subunits are specialized FaRLiP PBS components first reported in this work.

**Figure 1:**
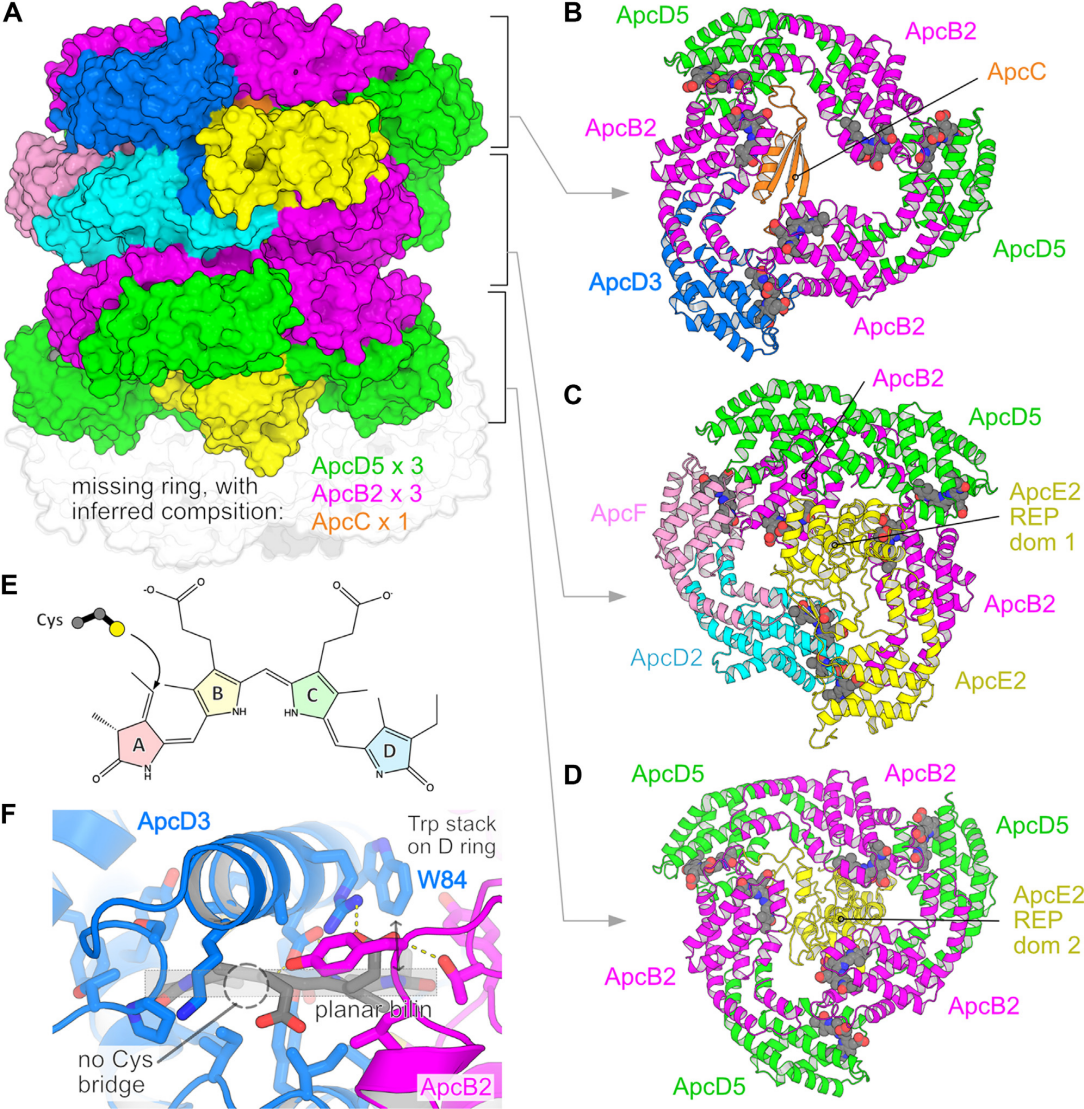
**Structure of the FaRLiP-adapted phycobilisome (Research Collaboratory for Structural Bioinformatics ID: 8UHE).***A*, overall structure of the phycobilisome, shown in surface representation. Note that the fourth ring of the complex is lost during purification, but its composition can be inferred. *B*–*D*, details of the organization of individual rings of the complex, shown in cartoon with bilin molecules shown as *gray spheres*. View is from the *top* of the complex as depicted in *A*. *E*, the structure of phycocyanobilin, showing the site of cysteine attachment. *F*, key structural factors shift the absorbance wavelengths of bilins in the α-allophycocyanin-binding sites to longer wavelengths, illustrated by the ApcD3 phycocyanobilin-binding site; note that an adjacent β-subunit (here ApcB2) contributes to the bilin-binding pocket. These include the stacking of a Trp residue on the pyrrole D ring, the binding of the bilin in an overall flat conformation, and in ApcE2 and ApcD3, the absence of a cysteine that usually is covalently linked to the pyrrole ring A. FaRLiP, far-red light photoacclimation.

Rather than using chlorophyll as the light-absorbing chromophore, PBPs use ring-opened derivatives of heme, known as bilins. PBPs primarily envelop bilins in a hydrophobic pocket found at the interface between αβ-heterodimers; typically, the bilin is also covalently linked to PBP through an enzyme-catalyzed thioether linkage to a conserved cysteine residue (Fig. 1*E*). The authors suggest that there are three main structural adaptations that red shift the absorbance spectra of these Apc paralogs. First, the α-subunits of the FaRLiP-specific paralogs of Apc bind a specific bilin, phycocyanobilin, in a conformation where the pyrrole ring A is more coplanar with the other rings than observed in their visible light–adapted paralogs (Fig. 1*F*). Second, the pyrrole ring D of all the α-subunit chromophores (except ApcA1) interacts with a tryptophan residue, rather than the more common tyrosine, a substitution previously shown to red shift the absorbance spectrum (9). Finally, ApcE2 and ApcD3 lack the cysteine that usually forms the thioether bridge to the chromophore. Because forming this covalent linkage reduces the terminal double bond, these unlinked bilins have an extended conjugated double-bond system, resulting in a further red shift in their absorbance spectrum. Of note, comparisons to previously characterized complexes suggest that ApcE2 and ApcD3 likely interact most directly with PSII; this enhanced red shift is therefore consistent with these two subunits serving as the terminal emitters of the complex (8).

The β-subunits (ApcB2 and ApcF) appear to be less adapted for far-red light absorbance than the α-subunits. Among the β-subunits, all except one are ApcB2; however, because the ApcC linker protein or the repeat domains of α-subunit ApcE2 form part of the bilin ring D pyrrole–binding pocket, each binding pocket has structural variation resulting in clear differences in the ring D conformation. The authors note that these conformational variations are also seen in the standard Apc core or else appear to arise from species-specific differences in sequence. Overall, the authors conclude that the β-subunit chromophores are unlikely to be important contributors to the red-shifted absorbance of the FaRLiP PBS core.

This work greatly advances our understanding of the FaRLip-adapted PBS core and shows how the FaRLiP-adapted α-Apc paralogs tune their bilin-binding sites to enhance the absorbance of longer wavelength light. In future work, it would be useful to capture the structure of the full core, with the fourth trimer still attached, perhaps by using a cross-linking strategy or by studying FaRLiP-adapted cores from a thermophilic cyanobacterial strain (such as *Leptolyngbya* sp. JSC-1).

## Conflict of interest

The author declares no conflicts of interest with the contents of this article.
